# Genetic variation of mitochondrial genes among *Echinococcus multilocularis* isolates collected in western China

**DOI:** 10.1186/s13071-017-2172-y

**Published:** 2017-05-30

**Authors:** Chuanchuan Wu, Wenbao Zhang, Bo Ran, Haining Fan, Hui Wang, Baoping Guo, Canlin Zhou, Yingmei Shao, Wei Zhang, Patrick Giraudoux, Jenny Knapp, Hao Wen, Ling Kuang, Jun Li

**Affiliations:** 1 Clinic Medicine Research Institute, The First Teaching Hospital of Xinjiang Medical University, Urumqi, China; 2 Collage of Veterinary Medicine, Xinjiang Agriculture University Urumqi, Xinjiang, 830000 China; 3 Centre for Clinic Echinococcosis, The First Teaching Hospital of Xinjiang Medical University, Urumqi, Xinjiang 830000 China; 4 Department of Hepatopancreatobiliary Surgery, Affiliated Hospital of Qinghai University, 251 Xining Road, 810000 Xi-Ning, Qinghai China; 5 Chrono-environmentLaboratory, University of Bourgogne Franche-Comté, UMR CNRS 6249, Besançon, 25000 France; 6 Institut Universitaire de France, Paris, 75015 France; 7 Department of Parasitology-Mycology, University Hospital of Besançon, Besançon, 25000 France

**Keywords:** *Echinococcus multilocularis*, Mitochondrial genes, *cob*, *nad*2, Genetic variation, Alveolar echinococcosis

## Abstract

**Background:**

Alveolar echinococcosis (AE) is a life-threatening human disease caused by *Echinococcus multilocularis* transmitted between rodents and dogs/foxes in the Northern Hemisphere. The study aims to identify the genetic variation of the parasite in AE patients from China.

**Methods:**

*E. multilocularis* isolates were collected from wild small mammals (*n* = 6) and AE patients (*n* = 56) from western China. Genomic DNA was extracted from different tissue samples including paraffin tissue blocks, ethanol fixed tissues and frozen tissues surgically removed. Two mitochondrial gene fragments (526 bp for *cob* and 474 bp for *nad*2*)* of *E. multilocularis* were amplified and sequenced.

**Results:**

The parasite fragment sequences of *cob* fragments from AE patients showed two haplotypes, and *nad*2 gene fragment sequences had four haplotypes. The gene sequences from *Microtus* sp. were 100% identical to the sequences of some isolates from AE patients. These haplotypes were distributed in both Qinghai and Xinjiang provinces. Alignment analysis with the sequences from the GenBank databases showed five genotypes including three Asian genotypes, one from Europe and one from North America.

**Conclusions:**

Most AE patients harbored the Asian genotype 1 which may be an indication of its relative frequency in the definitive hosts and the environment or of its pathogenicity to humans, which calls for further research.

## Background

*Echinococcus multilocularis* is a fox/dog tapeworm which causes human alveolar echinococcosis (AE). This zoonosis is one of the most lethal infections in humans worldwide [1, 2]. The tapeworm needs two hosts to complete its life-cycle: a definitive host such as a fox or a dog, and a small mammal intermediate host (vole, pika, zokor, hamster, jird etc.) [3]. The transmission of the disease is sustained in wildlife by the predator–prey relationship between the fox and small mammals. Domestic dogs play also an important role in the transmission of the disease to humans in central Asia including in western China [4–8]. Foxes or dogs are infected by feeding on small mammals infected with fertile *E. multilocularis* vesicles containing larval protoscoleces (PSCs). Once ingested, PSCs are released from their vesicles and develop into mature adult tapeworms producing eggs in the intestine of the carnivorous animal 28–35 days post-infection. The eggs are released through passing of dog/fox feces. Humans are infected as an intermediate host by accidently swallowing parasite eggs. However, humans are an epidemiological deadlock and are not involved in the life-cycle.

AE is more severe in humans than cystic echinococcosis which is caused by *Echinococcus granulosus*. The high mortality is due to the larval stage causing severe damages to the liver. About 95% of AE patients die within ten years in the absence of treatment [2]. Recently, many human AE cases were reported on the Qinghai-Tibet plateau [3, 6, 7, 9, 10]. It is estimated that western China including Xinjiang, Sichuan, Qinghai, Ningxia and Inner Mongolia, has 91% of total AE cases in the world [11].

Earlier studies have clearly shown that *E. multilocularis* is widely distributed in the Northern Hemisphere, and the large diversity of life-cycle patterns and host communities throughout its distribution range are currently described. Large genetic variations within the parasite species might be expected from such range and diversity as a response to various hosts and environmental adaptation. Paradoxically, little genetic variation was detected within *E. multilocularis* which was initially classified into two genotypes only, M1 (Europe) and M2 (China, Alaska and North America) based on four nucleotide substitutions out of 471 nt of mitochondrial NADH dehydrogenase subunit 1 gene [12]. However, based on sequence data of mitochondrial and nuclear DNA, further geographical distinctions were found with European, Asian and North American clades, and one unrelated haplotype from Inner Mongolia [13]. In fact, there are very few mitochondrial sequences of *E. multilocularis* from AE patients deposited in the GenBank database. In the present study, we sequenced two mitochondrial (mt) genes (fragments) from parasitic liver tissues of 56 human AE cases and six isolates of *E. multilocularis* collected from rodent intermediate hosts.

## Methods

### *Echinococcus multilocularis* samples

Three isolates of *E. multilocularis* were maintained in Mongolian jirds in our laboratory by transplantation of protoscoleces intraperitoneally for more than 10 years. Among them, Em-NX and Em-XJ were collected from *Microtus* spp. from southern Ningxia and Xinjiang, respectively. Em-A, an Alaskan strain was obtained from Professor Philip Craig, University of Salford, UK. Three *E. multilocularis* specimens were isolated recently from the livers of voles (*Microtus* sp.) from Yili, Xinjiang, China. The livers containing parasite tissues were fixed in 75% ethanol until use.

To identify the variation of *E. multilocularis* in humans, 56 AE tissue samples were collected from AE patients including 20 paraffin blocks for pathological tests, 12 liver tissues fixed in 70% ethanol and 24 liver tissues frozen at -80 °C. The patients were mainly from Xinjiang, Qinghai, Sichuan and Gansu provinces.

### DNA extraction

Genomic DNA was extracted from fresh protoscoleces of the three laboratory maintained isolates of *E. multilocularis*. The protoscoleces were aspirated from the parasite mass from Mongolian jirds. After 10 washes with PBS, about 100 protoscoleces were soaked in the genomic DNA extraction buffer-PrepMan ^TM^ Ultra Sample Preparation Reagent (Appied Biosystems, Foster City, USA) and then homogenized using a microtube mortar and pestle (Sigma, St. Louis, USA). The homogenate was heated at 95 °C for 10 min. The solution was directly used as a DNA template for PCR amplification after centrifugation at 12,000× *g* for 3 min.

Three sources of liver tissues from AE patients were used for molecular analyses including paraffin tissue blocks, ethanol fixed tissues and frozen tissues. To extract DNA from the blocks, the QIAamp DNA FFPE Tissue Kit (Qiagen, Hilden, Germany) was used. Briefly, the paraffin blocks containing liver leisure tissues of AE patients were cut into 8 μm thick sections. About 8–10 sectioned films were placed into a microtube containing 0.5 ml xylene. The extraction procedure was performed as recommended in the manufacturer protocols. The DNA sample was eluted from the column with 50 μl of water, and then stored at -80 °C for further use.

To extract DNA from ethanol fixed tissues (both human and rodent) or frozen tissues, about 0.1 g of tissue was cut into small pieces and then transferred into a microtube containing 387 μl lysis buffer (50 mmol/l Tris, 0.1 mol/l EDTA, 0.1 mol/l NaCl, pH 8.0), 10 μl 20% SDS, and 3 μl proteinase K (20 mg/ml). The tube was incubated at 56 °C overnight. After centrifugation at 13,000× *rpm* for 10 min, the supernatant was transferred into a new microtube containing 400 μl ethanol. The DNA was pelleted by centrifugation at maximum speed for 15 min then the pellet was washed with 600 μl 75% ethanol and centrifuged at maximum speed for 5 min. The pellet was resuspended with 50 μl of water and stored at -20 °C until used.

### PCR amplification of mitochondrial gene fragments

PCR was carried out in a 50 μl reaction mixture including 1 μl template DNA solution, 8 μl of dNTPs, 1 μl of each primer at 10 μM for *cob* and *nad*2 gene fragments (Table 1), 1 μl of Ex-Taq Polymerase (TAKARA BIO INC, Shiga, Japan), 5 μl of reaction buffer and added water to 50 μl. PCR reactions were performed for 35 cycles of denaturation (95 °C for 30 s), annealing (57 °C for 30 s) and extension (72 °C for 60 s). Two pairs of primers used were designed for the present study, in order to amplify the partial sequences of the mitochondrial genes cytochrome *b* (*cob*), and NADH dehydrogenase subunit 2 (*nad*2), respectively (Table 1). The size of the two gene fragments was 526 bp and 474 bp, respectively.

**Table 1:** Primers designed for amplification of the partial sequences of *Echinococcus multilocularis cob* and *nad2* genes by PCR

Primer	Sequence (5′-3′)
EmCob-F	GTTTAAACTGGTAGATTGTGGTTC
EmCob-R	CTCCACAGTAGAAATCACCATCA
EmNad2-F	GCGTTGATTCATTGATACATTGT
EmNad2-R	TAGTAAAGCTCAAACCGAGTTCT

### DNA sequence alignment and analysis

To sequence the gene fragments, we isolated PCR products from agarose gel (0.8%, w/v) by using the QIAquick Gel Extraction Kit (Qiagen, Hilden, Germany), and then inserted the DNA into an AT vector (pMD19-T Vector, TAKARA BIO INC, Shiga,. Japan)), according to these manufacture’s instructions. Clones containing inserts were sent to sequencing company for sequencing. The sequences obtained were aligned using Bioedit version 5 (http://www.mbio.ncsu.edu/bioedit/bioedit.html/) and compared to the reference sequences from the GenBank database.

Phylogenetic analysis was performed using MEGA 6 [14] and phylogenetic trees were constructed using neighbor-joining (NJ) method [Kimura's two-parameter distance analysis with a gamma shape parameter (a = 0.5)]. The robustness of phylogenetic trees was tested by bootstrapping with 1,000 replicates. A network of mtDNA haplotypes was illustrated by NETWORK 4.6.1.3 using statistical parsimony (http://www.fluxus-engineering.com/ sharenet.htm). We calculated the genetic distance among the subpopulations using MegAlign (www.dnastar.com/t-megalign.aspx) pairwise fixation index (Fst). Fst values close to 1 indicate extreme genetic differentiation between two subpopulations.

## Results

### Sequence variation among the isolates maintained in laboratory

**Fig. 1:**
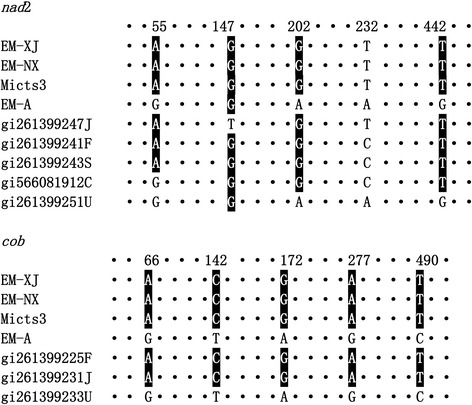
Alignment of single nucleotide polymorphisms among the three isolates of *E. multilocularis* maintained in laboratory animals (jirds) and three isolates from wild *Microtus* spp. (Mictus3) rodents. The top of each panel shows the position of the substitute mutations. *Abbreviations*: Em-XJ, *E. multilocularis* isolates were collected from Xinjiang; Em-NX, *E. multilocularis* isolates were collected from Ningxia; Mictus1-3, the three isolates collected recently from *Microtus*; Em-A, an *E. multilocularis* isolate was originally collected from Alaska, USA; *cob*, cytochrome b; *nad*2, NADH dehydrogenase subunit 2; C, Canada; F, France; J, Japan; S, Slovakia; U, USA. Only positions with mutations are listed in the figure, the identical nucleotides are represented by dots

The size of the amplified DNA fragments obtained from *E. multilocularis* isolates was 526 bp and 474 bp for *cob* and *nad*2, respectively (GenBank accession numbers: KT965443–KT965494; KY290762-KY290791). Figure 1 shows the substitution nucleotides of the two gene fragments amplified from the three isolates maintained in our laboratory and the three isolates we recently collected from wild rodents in Yili, Xinjiang. The comparison with the different haplotypes of the sequences deposited in the GenBank database showed that *E. multilocularis* is variable at the genomic level. It showed that *nad*2 sequences isolated from China were variable with a single base substitution compared with the sequences from France, Japan and Slovakia, two or more nucleotide substitutions from those from Canada and USA (Alaska). One of the *cob* haplotypes from China was identical to the sequences isolated from France and Slovakia, whereas, there are 5 substitutions compared with the sequences from Alaska, USA. The haplotype Em-A is identical to the sequence of the isolate collected from the St. Lawrence Island in Alaska (GenBank: AB461409), which is different from the two laboratory maintained isolates collected from China. The genetic divergence of *cob* and *nad*2 gene fragments between Em-NX/Em-XJ and Em-A ranged from 0.85 to 0.95%.

### Sequence analysis of *E. multilocularis* isolated from patients infected with alveolar echinococcosis (AE)

Liver tissue samples were collected from 56 AE patients by surgical removal. In total, 68 sequences were obtained from these samples including 32 *nad*2 gene sequences (including 24 from Xinjiang, 4 from Qinghai, 3 from Sichuan and 1 from Gansu); 36 *cob* gene sequences (23 from Xinjiang, 10 from Qinghai, one from Sichuan, Gansu and Beijing, respectively).

There are few *E. multilocularis* sequences from AE patients deposited in the DNA sequence databases, which may indicate the difficulty to obtain material or to amplify DNA fragments from surgical samples from AE patients. We extracted parasite DNA from three different sources of tissues. From the frozen tissues, 21 *cob* and 21 *nad*2 sequences were obtained from the 24 samples available (success rate: 87.5%). From paraffin blocks, we were successful in obtaining *nad*2 and *cob* sequences from 60% and 45% of the samples, respectively. From 12 ethanol-fixed samples, we obtained *nad*2 sequences only from 3 samples and *cob* sequences from 2 samples (Table 2).

**Table 2:** Tissue sources and *E. multilocularis cob* and *nad*2 sequences amplified from liver tissues surgically removed from alveolar echinococcosis patients

Tissue sources	Frozen	Paraffin	Ethanol	Total
Samples	24	20	12	56
*cob* sequences (%)	21 (87.5)	9 (45.0)	2 (16.7)	32
*nad*2 sequences (%)	21 (87.5)	12 (60.0)	3 (25.0)	36

**Fig. 2:**
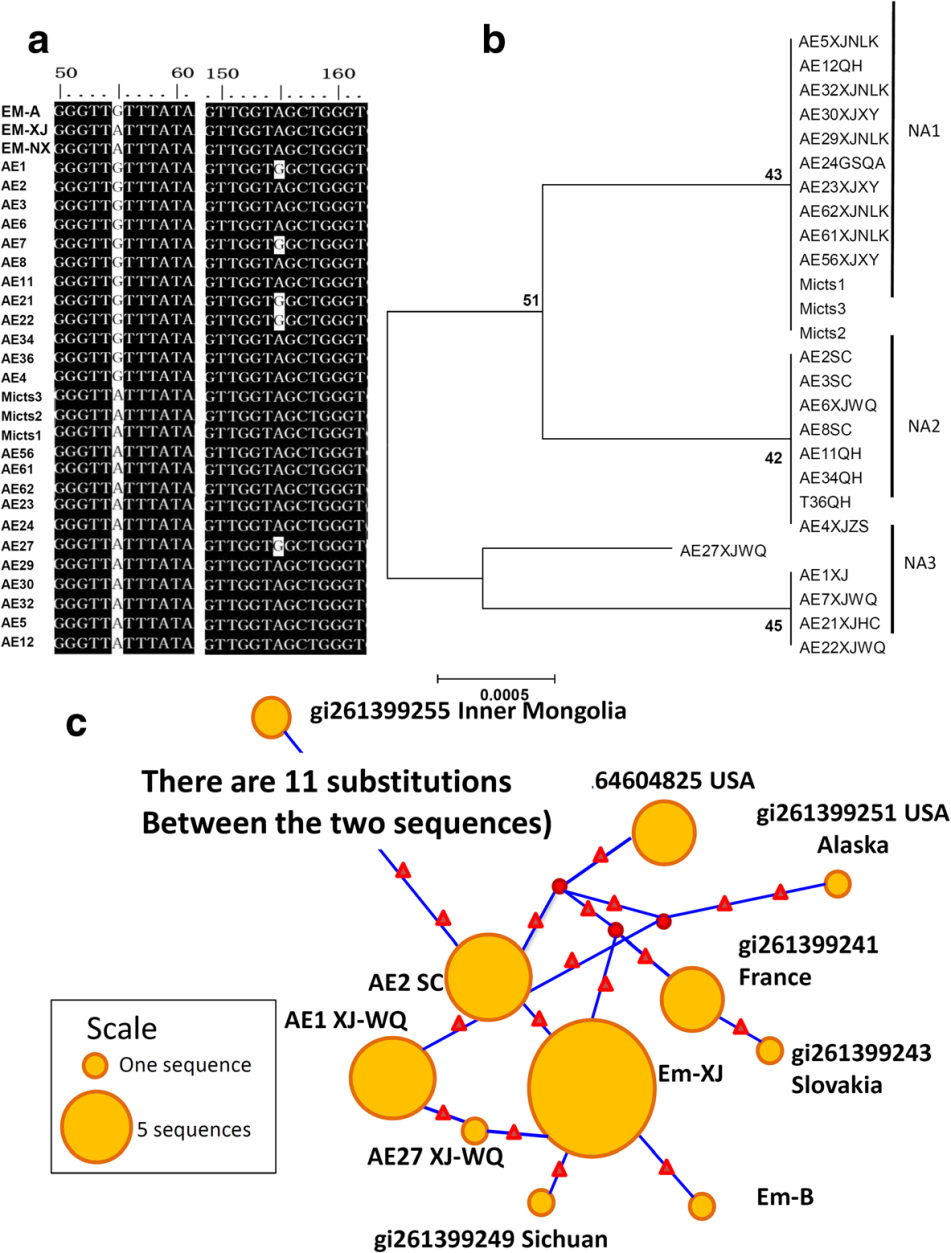
Neighbor-joining haplotype tree and parsimony network of *E. multilocularis nad*2. **a** Variation of *nad*2 sequences isolated from AE patients and rodents from China. **b** Phylogenetic tree from NJ analysis. Values on the nodes are bootstrap proportions (%). The scale-bar represents a divergence of 0.0005. **c** Parsimony network for *nad*2 combined with the sequences of *E. multilocularis* from the GenBank database. The circular icon represents a clade, and the small triangle icon represents a single base difference. NA1-3 represent 3 *nad*2 haplotypes. Liver samples were collected from AE patients from China including provinces of Qinghai (QH), Gansu (GS), Sichuan (SC), Xinjiang (XJ) with counties Wuqia County (WQ), Nileke County (NLK), Zhaosu County (ZS), Xinyuan County (XY), and Huocheng County (HC). Three isolates were collected from *Microtus* (Mictus1-3) in Yili valley in Xinjiang

The distance-based NJ analysis of *nad*2 gene sequences showed three clades referred to as NA1, NA2 and NA3 (Fig. 2a andb). The genetic divergence between NA1 and NA2 was 0.2%, that between NA1 and NA3 was 0.3%, The gap between NA1 and NA2 was not obvious with only one single nucleotide polymorphism (SNP) at position 55 (A/G). Compared to NA2 sequences, AE27 XJWQ had two substitutions at A55 to G55 and G155 to A155 (Fig. 2a) with genetic divergence of 0.4%.

We found some substitutions resulting in amino acid changes, *nad*2 155G in samples AE1, AE7, AE21, AE22 and AE27 is replaced by 155A in other sequences (Fig. 2a), which changes amino acid glycine (G) to serine (S). Whether the change is associated with the disease severity and pathological difference, and whether those differences are associated with different life-cycle patterns needs to be further elucidated.

The phylogenetic analysis based on the *nad*2 gene fragments showed that all the sequences isolated from AE patients were close to those isolates collected from rodents. In fact, some, such as sequences of AE2, AE12, AE24 and AE30, were identical to Em-XJ and Em-NX *nad*2 gene sequences. Alignment of these sequences combined with the sequences (*n* = 20) in the GenBank database shows that the amplified *nad*2 gene fragments (474 bp) contained 20 (4.2%) SNPs. Phylogenic analysis in a previous study showed that these sequences are divided into four clades, termed as Asian strain, European strain, North American strain and Alaska strain [13]. All of the sequences from AE patients belonged to the Asian strain (Fig. 2b).

The divergence value of the *nad*2 fragments of *E. multilocularis* collected from the three continents ranged from 0.4 to 1.3%. In addition, parsimony network analysis also supports the clade classification of *E. multilocularis* divided by their geographic locations (Fig. 2c).

**Fig. 3:**
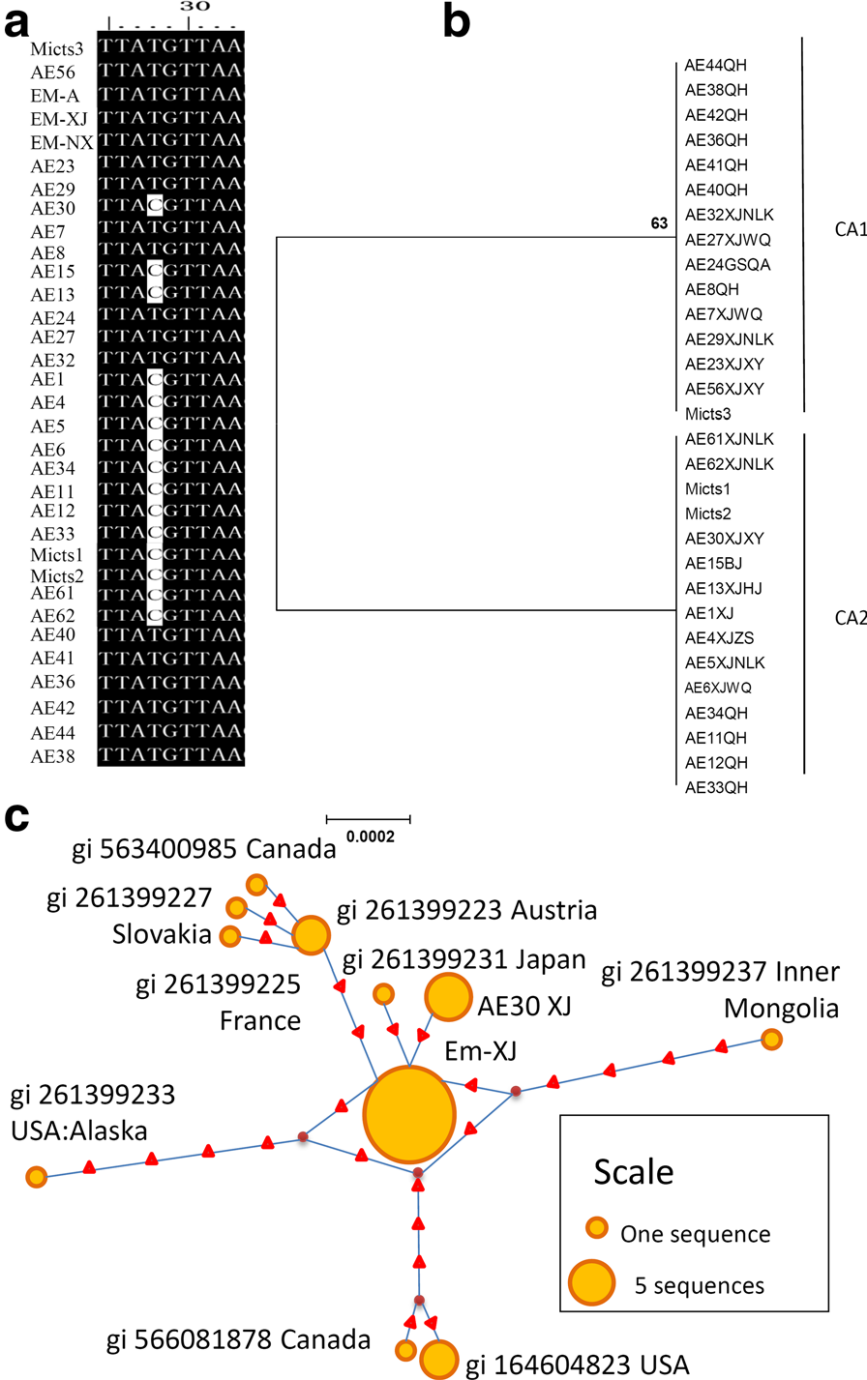
Neighbor-joining haplotype tree and parsimony network of *E. multilocularis cob*. **a** Variation of *cob* sequences isolated from AE patients and rodents from China. **b** Phylogenetic tree from NJ analysis. Values on the nodes are bootstrap proportions (%). The scale-bar represent a divergence of 0.0002. **c** Parsimony network for *cob* combined with the sequences of *E. multilocularis* from the GenBank database. The circular icon represents a clade containing identical sequences, and the small triangle icon represents a single base difference CA1 and CA2 represent 2 *cob* haplotypes. Liver samples were collected from AE patients from China including Beijing (BJ) and provinces of Qinghai (QH), Gansu (GS), Sichuan (SC), Xinjiang (XJ) with counties Wuqia County (WQ), Nileke County (NLK), Zhaosu County (ZS), Xinyuan County (XY), and Hejing County (HJ). Three isolates were collected from *Microtus* (Mictus1-3) in Yili valley in Xinjiang

The alignment of the 36 *cob* fragments showed two haplotypes with one nucleotide substitution at position 28(T/C) (Fig. 3a), which matches with the distance-based NJ phylogram analysis showing two genotypes, named CA1 and CA2 (Fig. 3b). The divergence between CA1 and CA2 haplotypes was 0.2%. However, the substitution causes no amino acid sequence change. The two haplotypes coexist in the two major Chinese AE endemic areas, Qinghai and Xinjiang.

The statistical parsimony networks for the *nad*2 and *cob* gene fragments illustrated in Figs. 2c and 3c showed that only the Asian genotype/clade is located in the center, the others are scattered. Fig. 2c shows that the *nad*2 sequences of the European genotype are among the American and Asian genotypes. The European clade contains four substitutions and the Asian clade has three substitutions. The strains from Alaska and Inner Mongolia have five substituted nucleotides compared to the Asian genotype sequences.

### The distribution of the genotypes in the world

**Fig. 4:**
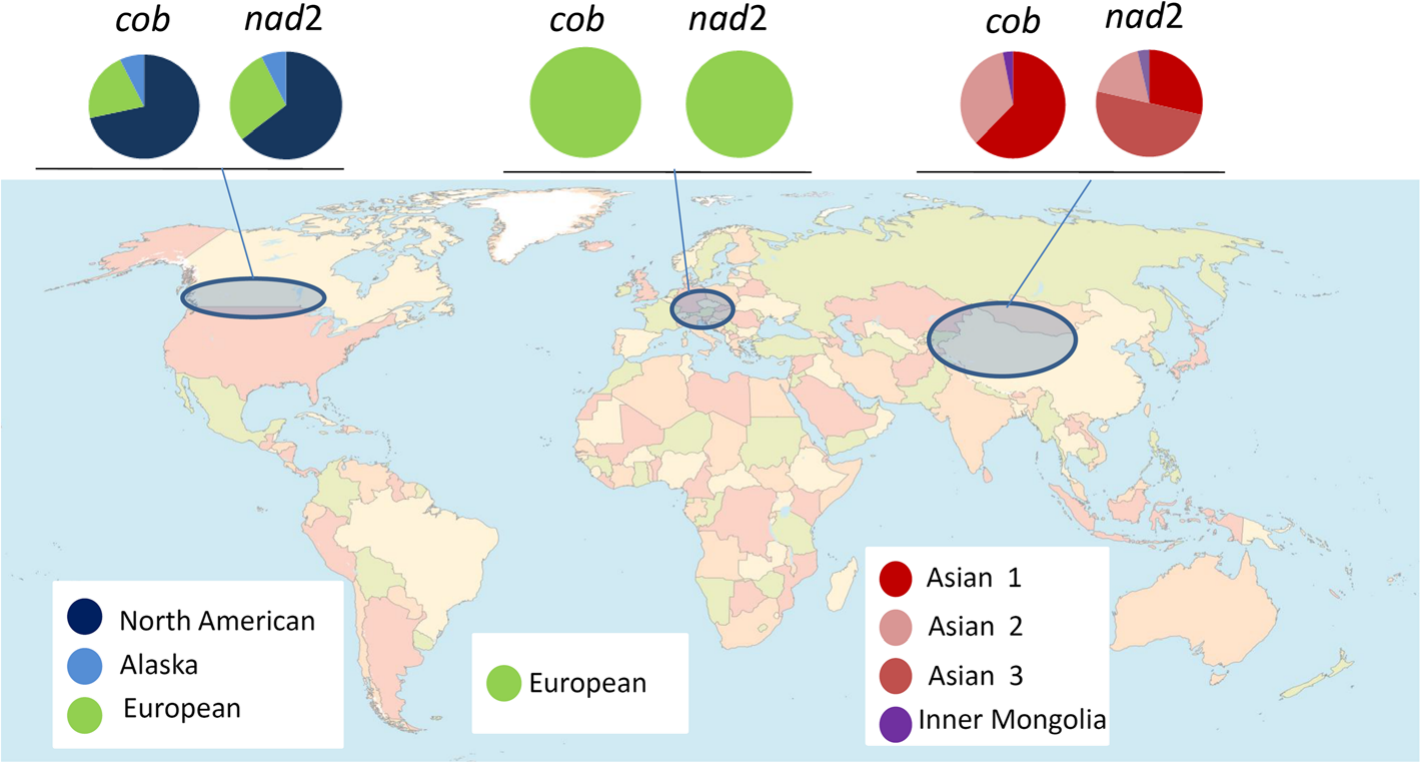
Worldwide distribution of *E. multilocularis cob* and *nad*2 haplotypes

Including our sequences, a total of 125 sequences from the GenBank database were employed to map the distribution of these genotypes of *E. multilocularis* worldwide (Fig. 4). The parasite exhibits different genotype patterns in different continents. In Europe, *nad*2 and *cob* are conserved, showing only one clade. In North America, there are three clades, the North American genotype (64–72%), the European genotype (21–29%) and the Alaskan genotype (7–8%). In Asia, *cob* gene sequences allowed us to distinguish two major genotypes.

## Discussion

The variations in the mitochondrial genes *cox*1 and *nad*1 have been used to determine the genotypes of *E. granulosus* (sensu *lato*) [12, 15, 16], which was classified into nine strains, now five species mainly according to their intermediate hosts, indicating that the host selection may play an important role in driving the genetic evolution. However, there is no evidence so far that such a selection is associated with the genomic variation of *Echinococcus*. In the study, we obtained 68 mitochondrial sequences for *E. multilocularis* from AE patients, including 36 *cob* and 32 *nad*2 gene sequences, offering basic information for identification of the relationship of host specificity and genetic variation. To our knowledge, this is the largest sequence dataset obtained from humans AE cases. We noted that *cox*1 of *E. multilocularis* has been used for identification of the variation within the genus *Echinococcus* [17]. However, in our study, only one *cox*1 sequence was generated from the human AE cases (data not shown).

Alignment and phylogenetic analysis of *nad*2 sequences from the patients showed four haplotypes in three clades, whereas the *cob* gene sequence analysis showed two haplotypes. It will be interesting to know whether the haplotypes are associated with different pathological patterns of AE which coexisted in two major Chinese AE endemic areas, Qinghai-Tibet Plateau and Xinjiang. However, in both areas, one genotype (Asian genotype 1) was predominant, which may indicate that this genotype of *E. multilocularis* is suitable to the rodent host species, environment and landscape, which impact the transmission of AE [1].

The phylogenetic tree and genetic divergence analysis showed that the European and Asian isolates are very close, indicating they had close evolutionary relationships, whereas, the North American and Alaskan genotypes are relatively far from the Asian clade (Figs. 2 and 3).

Sequence analysis of *E. multilocularis cob* and *nad*2 gene fragments showed relatively low genetic variation among the isolates from AE patients in western China. Most of the isolates are Asian genotype 1 which may be an indication of its relative high frequency as a source of infection to humans compared to other genotypes, either because it is much more frequent in definitive hosts close to humans (e.g. dogs) or because of a stronger pathogenicity to humans. These hypotheses need to be further elucidated.

## Conclusions

We collected *E. multilocularis* isolates from patients infected with alveolar echinococcosis from western China. The analysis of mitochondrial *cob* and *nad*2 gene fragments showed that the parasite exhibits variation at the genomic level with at least three genotypes in China.

## Abbreviations

AE: alveolar echinococcosis
*cob*: cytochrome *b* gene
*nad*2: NADH dehydrogenase subunit 2 gene
